# Determinants of survival of adolescents receiving antiretroviral therapy in the Centre Region of Cameroon: a multi-centered cohort-analysis

**DOI:** 10.1186/s12981-023-00584-2

**Published:** 2023-12-15

**Authors:** Nicholas Tendongfor, Joseph Fokam, Collins Ambe Chenwi, Fabrice Léo Tamhouo Nwabo, Armanda Nangmo, Njume Debimeh, Suzie Tetang Ndiang Moyo, Marie Patrice Halle, Anne-Esther Njom-Nlend, Paul Koki Ndombo, Alexis Ndjolo

**Affiliations:** 1 Faculty of Health Sciences, University of Buea, P.O. Box 63, Buea, Cameroon; 2 Chantal BIYA International Reference Centre for Research on HIV/AIDS Prevention and Management (CIRCB), PO Box 3077, Yaoundé, Cameroon; 3 Faculty of Medicine and Biomedical Sciences, University of Yaoundé I, Yaoundé, Cameroon; 4 Pediatric Department, National Social Welfare Centre, Essos Health Centre, Yaoundé, Cameroon; 5 Department of Internal Medicine, Douala General Hospital, Douala, Cameroon; 6 Faculty of Medicine and Pharmaceutical Science, University of Douala, Douala, Cameroon; 7 Higher Institute of Medical Technology, University of Douala, Yaoundé, Cameroon; 8 Mother and Child Centre, Chantal BIYA Foundation, Yaoundé, Cameroon

**Keywords:** HIV, Survival, Adolescent, ART, Rural, Urban, Cameroon

## Abstract

**Background:**

In spite of the global decreasing mortality associated with HIV, adolescents living with HIV (ADLHIV) in sub-Saharan Africa still experience about 50% mortality rate. We sought to evaluate survival rates and determinants of mortality amongst ADLHIV receiving antiretroviral therapy (ART) in urban and rural settings.

**Methods:**

A multi-centered, 10-year retrospective, cohort-study including ADLHIV on ART ≥ 6 months in the urban and rural settings of the Centre Region of Cameroon. Socio-demographic, clinical, biological, and therapeutic data were collected from files of ADLHIV. The Kaplan–Meier method was used to estimate survival probability after ART initiation; the log rank test used to compare survival curves between groups of variables; and the Cox proportional hazard model was used to identify the determinants of mortality.

**Results:**

A total of 403 adolescents’ records were retained; 340 (84%) were from the urban and 63 (16%) from the rural settings. The female to male ratio was 7:5; mean age (Standard deviation) was 14.1 (2.6) years; at baseline, 64.4% were at WHO clinical stages I/II, 34.9% had ≥ 500 CD4 cells/mm^3^, 91.1% were anemic, and the median [Inter Quartile Range] duration on ART was5.3 [0.5–16] years. The survival rate at 1, 5 and 10 years on ART was respectively 97.0%, 55.9% and 8.7%; with mean survival time of 5.8 years (95% CI 5.5–6.1). In bivariate analysis, living in the rural setting, non-disclosed HIV status, baseline CD4 count < 500 cells/mm^3^, not being exposed to nevirapine prophylaxis at birth and being horizontally infected were found to be the determinants of higher mortality with poor retention in care slightly associated with mortality. In multivariate analysis, living in rural settings, poor retention in care and anemia were independent predictors of mortality (p < 0.05).

**Conclusion:**

Although ADLHIV have good survival rate on ART after 1 year, we observe poor survival rates after 5 years and especially 10 years of treatment experience. Mitigating measures against poor survival should target those living in rural settings, anemic at baseline, or experiencing poor retention in care.

## Introduction

Human Immunodeficiency Virus/Acquired Immune Deficiency syndrome epidemic (HIV/AIDS) continues to be a major global public health issue. In 2022, an estimated 39 million people globally were living with HIV/AIDS [1]. In this same year, about 1.7 million adolescents between the ages of 10 and 19 were reported to be living with HIV worldwide, accounting for about four percent of all people living with HIV and about 10 percent of all new infections [2]. Of these, about 1.4 million (that is 85 per cent), live in sub-Saharan Africa, which remains the most affected region even in this adolescent population [2, 3]. The prevalence of HIV in Cameroon as of 2018, is 2.7% with 0.8% occurring among adolescents aged 15–19 [4]

Antiretroviral treatment (ART) has improved the survival of HIV-infected patients by inhibiting the viral replication, which in turn boosts the immune system through increased CD4 count, decreases in HIV/AIDS-associated manifestations, and minimizes the risk of opportunistic infections (OIs). However, taking into consideration non-adherence and drug resistance that may lead to poor responses, some patients might have suboptimal response to ART and remain at risk of HIV-associated morbidity and mortality [5]. Despite the overall decrease in HIV-related deaths in the general population, the proportion of deaths among adolescents have more than doubled during the period of 2000 to 2015 [6]. Indeed, adolescents living with HIV (ADLHIV) are the only age group with increasing HIV mortality in the current ART era, they have poor clinical and programmatic outcomes at each stage of the HIV care cascade, with increasing mortality rate as these adolescents grow older [7, 8].

HIV programs are increasingly recognizing adolescents as a critical age group. Nonetheless, adolescents continue to be underserved by current services across the HIV cascade. They have significantly inferior ART access and coverage, higher rates of loss to follow-up, poor adherence, and increased needs for psychosocial support and sexual reproductive health services [9]. These challenges are also associated with increased risks of virological failure and HIV drug resistance (HIVDR) emergence which is a threat to ART effectiveness in Sub-Saharan African health programs, including Cameroon [10, 11]. Of note, the prevalence of baseline HIVDR in pediatric population in Cameroon is around 50%, essentially driven by mutations associated with non-nucleoside reverse-transcriptase inhibitors (NNRTI) [12]. Furthermore, Cameroonian adolescents receiving ART were found with 34.2% rate of virological failure and over 90% rate of HIVDR among those experiencing treatment failure in the Centre region of the country [11, 12]. Hence adolescents are now recognized as a distinct population, and have been described as the ‘fulcrum’ and the center of the epidemic’ with different health requirements for children as compared to adults [9, 13]. In this context, identifying drivers of survival and mortality among adolescents, according to geographical localities in resourced-limited and HIV high-burdened countries, would be of paramount importance in defining gaps in the AIDS response. This study therefore aimed at assessing the survival rates of ADLHIV on ART and to identify the determinants of mortality in urban and rural settings of Cameroon.

## Methods

### Study design and settings

We carried out a multi-centred, 10-year retrospective cohort study among ADLHIV receiving ART from January 1st, 2010 to December 31st, 2019. Data was extracted from patients’ files sequentially from ART initiation till they were either lost to follow up, dead or till the end of the study period.

Our study population included ADLHIV having been on ART for at least 6 months in 6 HIV treatment centres in the urban and rural areas of the Centre Region of Cameroon. These were two approved HIV treatment centres in the urban setting namely (the Mother–Child centre of the Chantal BIYA Foundation and the National Social Welfare Hospital Yaoundé), and four HIV managements units in the rural setting (the Mbalmayo District Hospital, the Mfou District hospital, the Nkomo integrated health centre and the Bikop Catholic Medical centre). These sites were conveniently selected based on the geographical areas (urban vs. rural), availability of at least first line ART, experience in providing ART for at least 3 years and use of standard patient monitoring registers.

### Data collection

We collected data from adolescents’ medical records and ART files using a study-specific data collection form. The data extracted from records included: socio-demographic data (age, gender, parental status, area of residence, level of education, and disclosure of HIV to study participants). Clinical data (WHO clinical stage, mode of transmission, presence of opportunistic infection); biological data (baseline CD4 count at start, endpoint CD4 count, hemoglobin at start) were retrieved; therapeutic data (treatment regimen, loss to follow up, retention in care, cotrimoxazole prophylaxis, exposure to NVP prophylaxis for PMTCT). Anemia was defined as Hb level below 12 g/dl for female and 13 g/dl for male. The presence of opportunistic infection at any point in time during the study period was collected. Patients who had no contact, 90 days after their last missed pill appointment were considered as being lost to follow-up (LTFU) [14]. As such, we considered retention in care as patients known to be alive and receiving ART at the end of the follow-up in our study, excluding cases of reported deaths.

### Study participants, end points and outcome measures

Enrolled were all adolescents aged 10–19 years, followed up during the study period at one of our study sites, having been on ART for at least 6 months. Excluded were all participants who were transferred in or out from different centers during our study period, and participants with incomplete files. Our major end points were participants who died during our study period, or who were lost to follow-up LTFU. Our major outcomes were survival rates and mortality. For survival analysis, all 403 participants enrolled in the study were considered, while adjusting for those who were lost to follow-up.

### Statistical analyses and calculation methods

All data collected were checked at the end of each patient file to make sure all sections were adequately reported. We entered these data in Epi Info v7.2.2.6 and analyzed using statistical package for social science (SPSS) version 21. Kaplan–Meier method was used to perform survival analysis, to estimate survival probability after initiation of ART, and the log rank test used to compare survival curves between groups of variables. Cox proportional hazard model was used to identify key predictor of mortality. P-values < 0.05 were considered statistically significant.

### Ethical considerations

An ethical clearance was obtained from the Ethical Review Board of the Faculty of Health Sciences, University of Buea (Ref: 2020/1056-01/UB/SG/IRB/FHS) and regional delegation of Public Health of the Centre region, Cameroon (Ref: CE N^o^-3462-/CRERSHC/2020). Administrative approval was obtained from the directors of the various centers prior to the start of the study. The data collection tools were assigned codes and kept anonymous. All data collected during the study was kept strictly confidential and shared only with the investigators and management team of the various centers.

## Results

### Demographic and clinical characteristics of study population

Of 544 files, we had 403 eligible files of which 340 (84%) were from the urban setting and 63 (16%) from the rural setting. Majority of the study population 232/403 (57.6%) were females with F/M ratio of 7:5. Overall, mean age (± SD) was 14.08 (± 2.6) years. The median [IQR] duration on ART was 5.3 [0.5–16] years. Details on socio-demographic characteristics are presented in Table 1.

**Table 1:** Socio-demographic characteristics according to geographical settings

Characteristics		Urban n (%)	Rural n (%)	Total n (%)
Gender	Male	145 (42.7)	26 (40.1)	171 (42.4)
	Female	194 (57.3)	38 (59.9)	232 (57.6)
Age (years)	10–14	202 (59.6)	32 (50.0)	234 (58.1)
	15–19	137 (40.4)	32 (50.0)	169 (41.9)
	Mean ± SD	13.97 ± 2.52	14.72 ± 3.15	14.08 ± 2.64
Parental status	Orphan of both	53 (15.9)	3 (7.3)	56 (15.0)
	Orphan of mother	75 (22.6)	8 (19.5)	83 (22.2)
	Orphan of father	58 (17.4)	12 (29.3)	70 (18.7)
	Non orphan	147 (44.1)	18 (43.9)	165 (44.1)
Level of education	Primary	82 (28.1)	16 (32.7)	98 (28.7)
	Secondary	203 (69.5)	30 (61.2)	233 (68.3)
	Tertiary	4 (1.4)	1 (2.0)	5 (1.5)
	None	3 (1.0)	2 (4.1)	5 (1.5)
Disclosure of HIV to study participants	Yes	108 (36.1)	6 (20.7)	114 (34.8)
	No	191 (63.9)	23 (79.3)	214 (65.2)

Overall, 222/320 (69.4%) were in less-advanced WHO clinical stage (defined as WHO stages I/II); 75/215 (34.9%) of these adolescents had CD4 count ≥ 500 cell/mm^3^ at start of ART as compared to 54/75 (72%) at end point. When comparing the rate of CD4-recovery from baseline to end point, there was a significant recovery among those in urban settings (from 31.7% to 75.4%, p < 0.0002) as opposed to a slight decline in rural settings (from 55.2% to 50.0%, p = 0.7772). Retention in care on ART was 346/389 (88.9%), with a comparable trend between urban (88.6%) and rural (85.2%) settings. Similarly, the overall rate of lost to follow-up was 9.3%, with a comparable trend between urban (8.5%) and rural (13.3%) settings. Details on medical characteristics are presented in Table 2.

**Table 2:** Medical characteristics according to geographical settings

Characteristics		Urban n (%)	Rural n (%)	Total n (%)
Baseline CD4 count	≥ 500 cell/mm^3^	59 (31.7)	16 (55.2)	75 (34.9)
	< 500 cell/mm^3^	127 (68.3)	13 (44.8)	140 (65.1)
WHO clinical stage	stage I/II	179 (68.8)	43 (71.7)	222 (69.4)
	Stage III/IV	81 (31.2)	17 (29.3)	98 (30.6)
Endpoint CD4 count	≥ 500 cell/mm^3^	49 (75.4)	5 (50)	54 (72.0)
	< 500 cell/mm^3^	16 (24.6)	5 (50)	21 (28.0)
Retained in care	Yes	294 (88.6)	52 (85.2)	346 (88.9)
	No	36 (11.4)	8 (14.8)	47 (12.0)
Loss to follow up	Yes	28 (8.5)	8 (13.3)	36 (9.3)
	No	300 (91.5)	52 (86.7)	352 (90.7)
Anemia at start	Yes	129 (94.2)	24 (77.4)	153 (91.1)
	No	8 (5.8)	7 (22.6)	15 (8.9)
Opportunistic infection	Yes	42 (12.5)	5 (8.3)	47 (11.9)
	No	293 (87.5)	55 (61.7)	348 (88.1)
Cotrimoxazole prophylaxis	Yes	293 (92.4)	55 (90.2)	348 (92.1)
	No	24 (7.6)	6 (9.8)	30 (7.9)
Treatment regimen	First line^a^	306 (93.9)	52 (85.2)	358 (92.5)
	Second line^b^	20 (6.1)	9 (14.8)	29 (8.5)
Exposure to ^c^NVP prophylaxis for ^d^PMTCT	Yes	13 (7.6)	7 (1.8)	20 (9.5)
	No	158 92.4)	33 (98.2)	191 (90.5)
Mode of transmission	Vertical	236 (97.5)	29 (74.4)	265 (94.3)
	Horizontal	6 (2.5)	10 (25.6)	16 (5.7)

^a^NNRTI based regimen, ^b^PI/r based regimen, ^c^Nevirapine, ^d^Prevention of mother to child transmission

### Survival outcome of HIV infected adolescents after initiation of ART

**Fig. 1:**
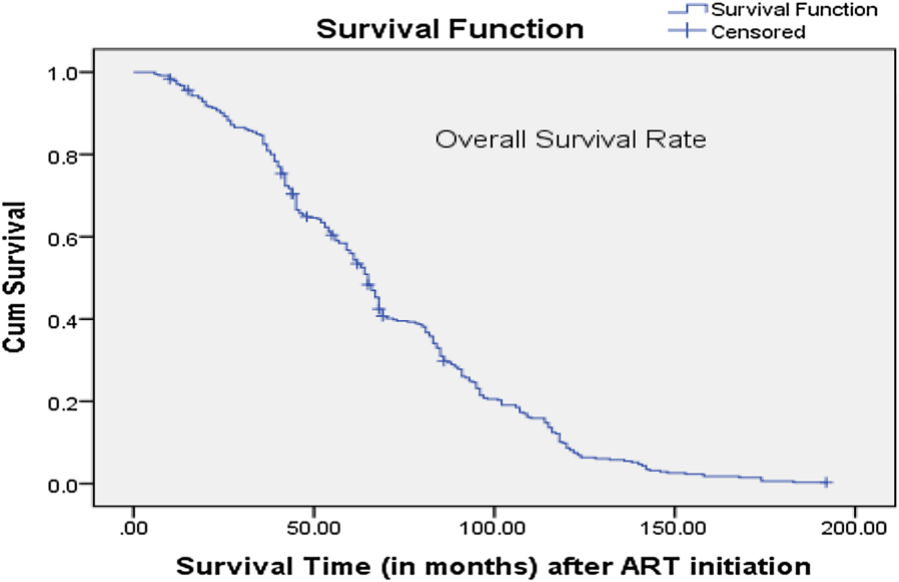
Overall survival curve (n = 403): the figure shows the overall survival curve for the adolescents enrolled in the study

The mean survival time was 69.7 months (95% CI 65.8–73.5) and overall cumulative probabilities of survival respectively at 1, 5 and 10 years after ART initiation were 97%, 55.9% and 8.7%. Of note, there was about 50% decline in survival after about 70 months on ART (see Fig. 1).

### Survival rate according to socio-demographic/clinical features

**Fig. 2:**
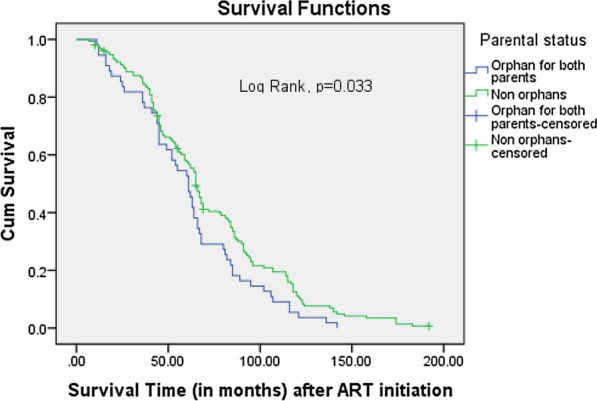
Survival curve with respect to parental status (being orphan for both parents versus non-orphans): the figure shows the survival curves for adolescents who were orphans for both parents (in purple), in comparison to those who were not orphans

**Fig. 3:**
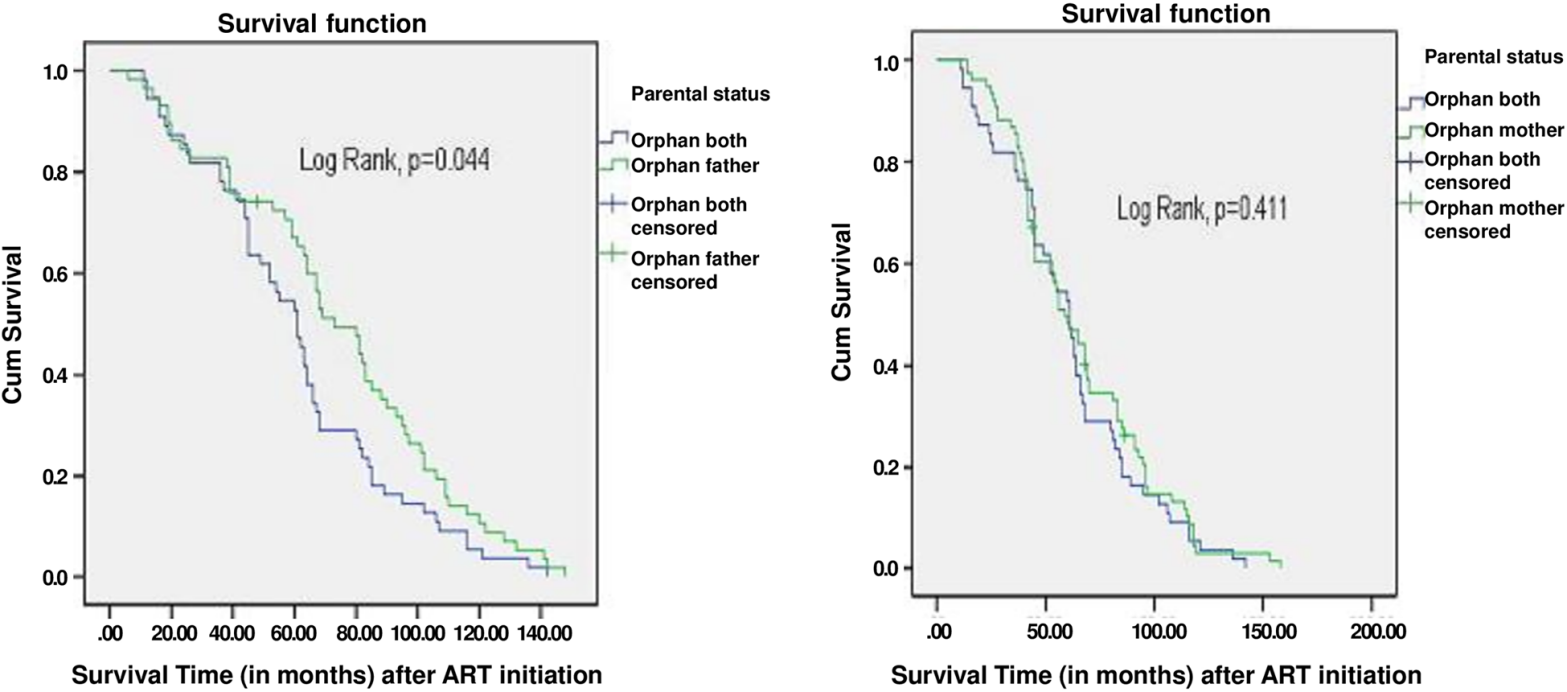
Survival curve between orphans both parents and orphan for a single parent (father and mother respectively): on the left, are the survival curves for adolescents who were orphans for both parents (purple) as compared to those who were paternal orphans (green) and on the right are the survival curves for adolescents who were orphans for both parents (purple) as compared to those who were maternal orphans (green)

According to orphan hood, adolescents who were non-orphans had a significantly higher survival rate as opposed to those who were orphans; Log Rank p = 0.033 (Fig. 2). Using orphans of both parents as a reference (Fig. 3), there was a similar survival rate with maternal orphans (Log Rank p = 0.411), as opposed to a significantly higher rate of survival among paternal orphans (Log Rank P = 0.044).

The survival time was significantly longer among those in the urban settings as compared to those in rural settings (mean survival time 72.9 versus 51.0 months respectively, p < 0.0001), which goes along with the hazard of mortality being 6 times higher. In addition, the survival time was longer among those with a disclosed HIV status (76.9 versus 67.8 months respectively, p = 0.04), as shown in Tables 3, 4.

**Table 3:** Mean survival time with respect to socio-demographic characteristics

Characteristics		Mean survival time in months (95% CI)	p-value (Log Rank)
Gender	Male	72.2 (66.5–77.9)	0.444
	Female	67.9 (62.7–73.0)	
Age (years)	10 – 14	72.8 (68.1–77.6)	0.185
	15 – 19	65.0 (58.7–71.4)	
Geographical setting	Urban	72.9 (68.9–77.0)	0.0001
	Rural	51.0 (40.2–61.8)	
Level of education	Primary	66.3 (52.4–73.1)	0.972
	Secondary	65.2 (60.7–69.7)	
	Tertiary	61.4 (29.3–93.5)	
	None	58.8 (11.3–106.2)	
Disclosure of HIV to study participants	Yes	76.9 (70.0–83.9)	0.040
	No	67.8 (62.9–72.6)	
Parental status	Orphan of both	61.5 (53.0–70.0)	0.084
	Orphan of mother	66.1 (58.6–73.6)	
	Orphan of father	73.7 (64.2–83.3)	
	Non orphan	72.6 (66.3–78.9)

**Table 4:** Mean survival time with respect to medical characteristics

Characteristics		Mean survival time in months (95% CI)	p-value (Log Rank)
Baseline CD4 count	≥ 500 cell/mm^3^	87.3 (77.3–97.4)	0.001
	< 500 cell/mm^3^	66.9 (61.3–72.4)	
WHO clinical stage	Stage I/II	64.0 (58.9–69.1)	0.769
	Stage III/IV	62.3 (53.3–71.2)	
Retained in care	Yes	89.7 (65.8–113.5)	0.057
	No	68.7 (64.8–72.6)	
Opportunistic infection	Yes	61.1 (51.1–71.1)	0.080
	No	71.1 (67.0–75.3)	
Cotrimoxazole prophylaxis	Yes	68.2 (64.2–72.1)	0.351
	No	75.6 (59.8–91.4)	
Treatment regimen	First line^a^	68.6 (64.5–72.7)	0.346
	Second line^b^	81.2 (70.2–92.2)	
Exposure to ^c^NVP prophylaxis for ^d^PMTCT	Yes	83.2 (64.2–102.2)	0.010
	No	60.3 (55.4–65.2)	
Mode of transmission	Vertical	69.9 (65.3–74.5)	0.001
	Horizontal	40.2 (23.3–57.0)	

^a^NNRTI based regimen, ^b^PI/r based regimen, ^c^Nevirapine, ^d^Prevention of mother to child transmission

### Determinants of mortality in the study population

We observed a total of 14 deaths, 3 (4.7%) in the rural region and 11 (3.2%) in urban region. With respect to socio-demographic parameters, both the geographical settings (living in rural, p = 0.0001) and non-disclosure of HIV status (p = 0.044) were associated with a higher risk of mortality. Following multivariate analysis, only the geographical setting (living in rural: hazard ratio: 6.5; p = 0.0001) was an independent factor of mortality in our study population (Table 5).

**Table 5:** Determinants of mortality

Characteristics		Univariate Analysis		Multivariate Analysis	
		Hazard Ratio (95% CI)	P-value	Hazard Ratio (95% CI)	P-value
Gender	Male	1		–	–
	Female	1.081 (0.874–1.336)	0.473	–	–
Age in years	10–14	1		–	–
	15–19	1.167 (0.939–1.450)	0.163	–	–
Geographical setting	Urban	1		1	
	Rural	1.703 (1.268–2.286)	0.0001	6.521 (3.776–11.261)	0.0001
Level of Education	Primary	1		–	–
	Secondary	1.039 (0.810–1.332)	0.765	–	–
	Tertiary	1.190 (0.483–2.933)	0.705	–	–
	None	1.121 (0.411–3.058)	0.823	–	–
Disclosure of HIV to study participants	Yes	1		–	–
	No	1.280 (1.007–1.627)	0.044	–	–
Parental Status	Orphan	1.200 (0.963–1.495)	0.105	–	–
	Non orphan	1		–	–
Baseline CD4 count	≥ 500 cell/mm^3^	1		–	–
	< 500 cell/mm^3^	1.695 (1.230–2.337)	0.001		
WHO clinical stage	Stage I/II	1		–	–
	Stage III/IV	1.039 (0.801–1.349)	0.773	–	–
Retained in care	Yes	1		1	
	No	1.813 (0.962–3.418)	0.066	2.399 (1.156–4.979)	0.019
Anemia at start	Yes	1		1	–
	No	0.373 (0.204–0.684)	0.001	0.207 (0.067–0.632)	0.006
Opportunistic infection	Yes	1.333 (0.961–1.850)	0.086	–	–
	No	1		–	–
Treatment Regimen	First line	1.202 (0.815–1.771)	0.354	–	–
	Second line	1		–	–
Cotrimoxazole prophylaxis	Yes	1		–	–
	No	0.820 (0.536–1.254)	0.360	–	–
Exposure to NVP prophylaxis for PMTCT	Yes	1		–	–
	No	1.845 (1.134–3.003)	0.014	–	–
Mode of Transmission	Vertical	1		–	–
	Horizontal	2.575 (1.464–4.530)	0.001	–	–

With respect to medical parameters, when assessing factors potentially associated with risk of mortality, univariate analysis revealed that baseline CD4 less than 500 cell/mm^3^ (p = 0.001), non-exposure to nevirapine prophylaxis for PMTCT (p = 0.014), horizontal mode of HIV transmission (0.001) and anemia (p = 0.001) appeared as determinants of mortality among these adolescents. Following multivariate analysis, only poor retention in care (p = 0.019) and anemia at the start of ART (p = 0.006) were independent predictors of adolescent mortality (see Table 5).

## Discussion

With the goal to set-up measures to limit the occurrence of mortality among ADLHIV, our study aimed at assessing the rate of survival and determinants of mortality among ADLHIV receiving ART in urban and rural settings of Cameroon. Overall, the mean survival time was 69.7 months with a good survival rate at 1 year (97%), which significantly reduced at 5 years (55.9%) and 10 years (8.7%). These results are different from other findings, which suggest poorer survival rates soon after ART initiation. Lumbiganon et al*.* in 2011 described poor rates of survival soon after ART initiation, with a higher 5-year survival rate of 91.7% [15]. This higher survival rate could be due to the predominantly child population in their study, consistent with findings in our context at this time [7, 21]. The overall mean survival time in our study is not too different from findings by Arage et al. in Ethiopia (91.6 months). This slight difference can however be explained by the fact that, Arage et al. worked on a much younger population (2 months to 14 years) [5]. It is worth noting that Fokam et al. showed better treatment outcomes in children as compared to adolescent before 2019, therefore explaining the lower survival rates in our adolescent population at this time [16]. Furthermore, a number of factors and evidence concerning ADLHIV in the Cameroonian context could explain the high mortality rates observed in our study. A study in conducted by our same team (Fokam et al.) in 2017 showed that in the long term, ADLHIV were faced with risks of adherence problems and viral rebound [17]. Furthermore, long periods of treatment with low genetic barrier ARV drugs in this resource limited setting, lead to high rates of virological failure and HIV drug resistance in this target population. To add, the same team in 2019 showed declining rates of virological success after 36 months of treatment on NNRTI regimes, driven by poor adherence and probably emergence of drug resistance mutations to NNRTI which have a low genetic barrier. In 2020, Dambaya et al. described rates of 52.6% for overall drug resistance in treatment naïve patients (NNRTI resistance consisting 31.6%), with rates of NNRTI resistance as high as 90%, similar to findings by Fokam et al. in 2021 [11, 12]. Lastly, the majority of our adolescents were either single or double parent orphans, with most of them not knowing their status (non-disclosure). All these above-mentioned points could therefore explain the high mortality in our study, especially long after initiation (significantly after 5 years in our study). This therefore again calls for specific/adapted monitoring strategies in this population as they grow older.

Adolescents living in the rural setting had about six-fold risk of mortality as compared to those in urban settings. This is in line with challenges on timely access to ART and retention in the continuum of care in these rural settings [18]. To add, rural settings are faced with problems in administering HIV management and care, such as poor adherence to ART program and viral load coverage as described by Fokam et al. in 2020 [19]. This therefore calls for the effective implementation and scale up of better strategies such as community dispensation of ART and even HIV services (such as viral load testing) in rural areas, so as to fill this gap, while also mitigating the high patient-to-staff ratio (i.e. higher workload) and distances from healthcare centers in rural settings [12, 22]. It is worth noting that, community dispensation of ART was initiated in Cameroon in 2016, but needs to be expanded from urban areas (where the large majority of patients and treatment centers are located) to rural settings, so as to improve the availability of ART in the rural setting [20].

Elsewhere, poor retention in care was associated with higher rates of mortality as adolescents with poor retention in care had 2.4 times the risk of mortality as compared to those with good retention in care. These results are not surprising, as the benefits of retention in care cannot be overemphasized. In 2019, Ulloa et al. showed a marked decrease of mortality in patients who were successfully retained in care. Key interventions are therefore needed to solve this problem. A patient-centered approach, (by leveraging on the adolescent’s strengths for positive encouragements and by educating adolescents on healthy lifestyles to improve treatment adherence), might help [21–23]. This requires emphasis on specific healthcare needs of adolescents, the setting-up of adolescent- and youth-friendly health services, adolescent HIV guidelines/policies, peer mentoring with the real-life example of RECAJ + in Cameroon and other key adolescent-specific issues (i.e. intrinsic factors such as adolescent behaviors) [24].

Lastly, adolescents who were anemic had about fivefold increased risk of mortality, as compared to non-anemic adolescents. This is consistent with findings by Harding et al.in the United States, who described anemia as a predictor of mortality [25]. Also, Biru et al., reported anemia as being a predictor of attrition in children [26]. While continuing monitoring of anemia under zidovudine-containing regimens is essential, investigating the effect of advanced HIV disease at diagnosis or other contributing pathology on mortality risk is needed [27]. Given the relatively low cost of assessing anemia, this event can be used frequently to identify high-risk ADLHIV in such settings [27, 28].

Despite the successful completion of our study objectives, missing or poor records for some participants limit the statistical strength we would have loved to have. Nonetheless, the overall high sample size of the target population, the exhaustive sampling strategy in the study sites and our study design as a whole were potential strengths of our study. As perspectives, assessing the impact of delayed ART initiation on health outcomes, particularly among adolescents with congenital HIV-infection, is a research gap that we would love to cover in subsequent studies.

## Conclusion

There is a significant decreasing survival among ADLHIV on ART in the Centre region of Cameroon. Of note, ADLHIV on ART in the rural setting experience higher risk of mortality as compared to their peers in the urban settings. Irrespective of geographical settings, poor retention in care and anemia might be predictors of mortality among ADLHIV on ART. Thus, setting-up adolescent-friendly centers, peer mentoring and community provision of ART could substantially improve survival in Cameroon.

## Data Availability

Data supporting the findings are fully available in the results, in the tables and figures of the manuscript.

## Abbreviations

ADLHIV: Adolescent living with HIV
AIDS: Acquired Immuno-deficiency Syndrome
ART: Antiretroviral therapy
CD4: Cluster differentiation 4
HAART: Highly Active Antiretroviral Treatment
HIV: Human immuno deficiency virus
HIVDR: HIV drug resistance
LTFU: Loss to follow up
NNRTIs: Non-nucleoside reverse transcriptase inhibitors
NRTIs: Nucleoside reverse transcriptase inhibitors
NVP: Nevirapine
OI: Opportunistic infections
PI: Protease inhibitor
PMTCT: Prevention of mother-to-child transmission
SSA: Sub-Saharan Africa
UNAIDS: Joint United Nations programme on HIV/AIDS
UNDP: United Nations Development Programme
WHO: World Health Organization
